# Purification of High Salinity Brine by Multi-Stage Ion Concentration Polarization Desalination

**DOI:** 10.1038/srep31850

**Published:** 2016-08-22

**Authors:** Bumjoo Kim, Rhokyun Kwak, Hyukjin J. Kwon, Van Sang Pham, Minseok Kim, Bader Al-Anzi, Geunbae Lim, Jongyoon Han

**Affiliations:** 1 Department of Electrical Engineering and Computer Science, Massachusetts Institute of Technology, 77 Massachusetts Avenue, Cambridge, MA 02139, USA; 2 Department of Mechanical Engineering, Massachusetts Institute of Technology, 77 Massachusetts Avenue, Cambridge, MA 02139, USA; 3 Department of Mechanical Engineering, Pohang University of Science and Technology, San31, Pohang, Gyeongbuk, 790784, Republic of Korea; 4 Singapore-MIT Alliance for Research and Technology (SMART) Centre, Singapore; 5 Environmental Technology Department and Management, College of Life Sciences, Kuwait University, Kuwait; 6 Department of Biological Engineering, Massachusetts Institute of Technology, 77 Massachusetts Avenue, Cambridge, MA 02139, USA

## Abstract

There is an increasing need for the desalination of high concentration brine (>TDS 35,000 ppm) efficiently and economically, either for the treatment of produced water from shale gas/oil development, or minimizing the environmental impact of brine from existing desalination plants. Yet, reverse osmosis (RO), which is the most widely used for desalination currently, is not practical for brine desalination. This paper demonstrates technical and economic feasibility of ICP (Ion Concentration Polarization) electrical desalination for the high saline water treatment, by adopting multi-stage operation with better energy efficiency. Optimized multi-staging configurations, dependent on the brine salinity values, can be designed based on experimental and numerical analysis. Such an optimization aims at achieving not just the energy efficiency but also (membrane) area efficiency, lowering the true cost of brine treatment. ICP electrical desalination is shown here to treat brine salinity up to 100,000 ppm of Total Dissolved Solids (TDS) with flexible salt rejection rate up to 70% which is promising in a various application treating brine waste. We also demonstrate that ICP desalination has advantage of removing both salts and diverse suspended solids simultaneously, and less susceptibility to membrane fouling/scaling, which is a significant challenge in the membrane processes.

The majority of technology development efforts for water desalination so far have focused on the treatment of seawater or brackish ground water, while brine (water with higher salinity than 35,000 mg/L TDS) has received relatively little attention. Due to the presumably smaller market needs and lack of cost-effective treatment technology options, brine water has been considered as the waste product of desalination and typically disposed without further treatment. However, the state of affairs related to brine water has recently changed significantly, with more attention paid to the issue of produced water^1^ from shale oil/gas development and brine concentrated waste^2^ from seawater desalination plants. Especially in the US, proper management of produced water, which is the contaminated waste water resulting from hydraulic fracturing, has emerged a crucial task for shale oil and gas developers, for mitigating potential risk for environment and human health^3^. Brine desalination in unconventional oil and gas development would be highly desirable, as it reduces the use of fresh water necessary for hydraulic fracturing, and also minimizes wastewater disposal by deep-well injection, which is increasingly costly due to lack of available disposal wells and the need for transporting produced water^4^. In addition, massive and continued release of brine waste from seawater desalination plants in gulf coast countries are posing long term environmental risks, because elevated salinity and chemicals used to reduce biofouling could be harmful on the marine environment of relatively enclosed Persian gulf sea^2^,^5^.

For the purpose of brine desalination, reverse osmosis (RO), considered as the most economical technology in seawater treatment, is not energy- and cost-efficient in ultra-high salinity ranges (>45,000 mg/L of TDS)^6^,^7^. It also requires considerable pretreatment for maintenance and preventing membrane fouling, thereby rendering it unattractive for heavily contaminated produced water brine. Instead, thermal distillation and vapor compression are current technology options in high salinity brine treatment. Vapor compression, such as Mechanical Vapor Recompression (MVR) which is currently used in produced water desalination, can produce high purity water from feedwater of up to 125,000 mg/L of TDS, but requires high capital expense and therefore necessitates substantial water cost for complete desalination, ranges roughly $3.50–6.25/bbl ($22–39/m^3^, levelized cost)^7^,^8^,^9^. This significantly adds to the overall brine treatment cost, making the treatment of brine and produced water difficult to justify economically in many situations. In addition, both RO and distillation processes are designed for complete salt removal, a partial brine desalination process is worth consideration as optimal solutions. This is because (1) the main purpose of brine treatment is often lowering the salinity to a certain degree for reuse or discharge^10^, and (2) one can consider other economical desalination options (*e.g.* reverse osmosis), once salinity of brine waste is reduced to near seawater salinity^11^.

Electrodialysis is an electrically-driven membrane desalination technology that removes anion through anion exchange membrane (AEM) and cation through cation exchange membrane (CEM), as shown in Fig. 1a. Although electrodialysis is a mature technology, there are still new ideas derived from electrodialysis, such as reverse electrodialysis (RED)^12^,^13^ to generate power from different salt concentration, and shock electrodialysis^12^,^13^ to utilize overlimiting current for desalination by microporous structure near the membrane. On the other hand, electrical membrane desalination (*i.e.* electrodialysis) has often been considered to be competitive only for low salinity water treatment^14^, even though low resistance of high concentration brine should provide favorable conditions for any electrical desalination process. Except for a few works examining the electrodialysis with high salinity water^15^,^16^, there has been no systematic investigation on the merits of electrical membrane desalination with high salinity water beyond seawater level. However, recent studies by Lienhard and coworkers^11^,^17^ re-evaluated electrodialysis as an economically viable technology option for high salinity water treatment although they have left several issues (*e.g.* process optimization, membrane fouling) unaddressed. Previously, our group has suggested novel ICP desalination process, where both dilute and concentrate streams are generated between membranes and then separated by bifurcating channels at the end^18^ (Fig. 1b). Since majority of ions existed in salty water sources (*e.g.* seawater, produced water) are sodium and chloride (*D*_*Na*+_ = 1.33, *D*_*Cl−*_ = 2.03[10^−9^ m^2^ s^−1^]), ICP utilizing CEMs can enhance salt removal ratio up to 20% compared to electrodialysis under constant current applied, along with other advantages compared with related electrodialysis technique^19^. Motivated by this analysis, we continue to develop and customize ICP desalination for high salinity water treatment further. In this work, we show that ion concentration polarization (ICP) desalination, which utilizes two ion exchange membranes with the same polarity, can be a viable solution for highly contaminated brine treatment. Based on experiments and numerical simulation, we demonstrate an optimized multi-staging strategy to minimize the overall water cost. We also demonstrate economic viability of multi-stage ICP desalination for high salinity water treatment (~100,000 mg/L of TDS) with detailed cost analysis. Furthermore, unique advantages of ICP desalination over the conventional electrodialysis, such as processability of suspended solids and operation stability/durability, are demonstrated.

## Material and Method

### Materials

0.1–1.7 M NaCl solutions (5,800–100,000 mg/L of TDS) were used as feed solution while Na_2_SO_4_ solutions with same normality as feed solution were used as rinsing solution in the electrode channels. 1.5 μM Alexa Fluor 488 (Invitrogen, Carlsbad, CA) was added to visualize ICP phenomenon. We used *E. coli* K-12 strains (MG1655) that constitutively expresses green fluorescent protein to visualize bacterial removal in the microfluidic device. Crude oil (Greene, Co., PA) and 0.1 M NaCl solutions were mixed with volumetric ratio of 1:50 followed by sonication for 2 hours. For tracing fluid, small amount of fluorescent color coding red dye (Kingscote Chemicals, Inc., OH) was added to the solution with volumetric ratio of 1:1000. 5 wt% of pH indicator dye (Micro Essential Laboratory, Inc., NY) was used for pH visualization in the microfluidic channel. Natural seawater (Revere beach, MA) was directly used after general precipitation without any pre-filtration.

### Device fabrication

Experiments presented in this paper were mostly conducted in PDMS (Polydimethylsiloxane)-based microfluidic model system^20^,^21^ in order to visualize fluidic behaviors and trace the concentration distribution in microfluidic detail. (Figure 2a,b) Distance between membranes is 1.5–2.0 mm with 0.2–0.6 mm depth and 10–100 mm length. Fumasep FTCM-E, FTCM-A (FuMA-Tech GmbH, Germany), and carbon paper (Fuel Cell Store, Inc., Boulder, CO) are used as CEM, AEM, and electrode respectively. Plastic-based ICP/ED device was assembled by CEMs, AEMs (used in electrodialysis exp. only), carbon electrode, and laser-cut plastic components with 3 mm channel depth while other miscellaneous dimensions were kept same as microfluidic devices. After fabrication, all membranes were put in demineralized water or process solution for at least 48 hours at room temperature before experiments.

### Experiment

The negative pressure-driven flow was generated by syringe pump (Harvard apparatus, PHD 2200) and constant current and voltage were applied by current-voltage source measurement unit (Keithley 236, Keithley Instruments, Inc., OH). In order to exclude electrode and reaction overpotential occurred in the rinsing channel and measure voltage drop per cell solely, Ag/AgCl electrodes (A-M Systems, WA) were penetrated to the fluidic channels vertically and connected to the digital multimeters (Keithley model 2000 Digital Multimeter, Keithley Instruments, Inc., OH). (Figure 2c) Most experiments were done on the microscope (Olympus, IX-71) and conductivity of output flow was traced by connecting a flow-through conductivity probe (Microelectrode Inc., NH) to quantify the salt removal. (Figure 2d) Analysis of quantitative removal of monovalent and divalent cations was performed by CCD-based ICP-AES instrument (ACTIVA-S, Horiba, Ltd., Japan).

### Numerical modeling

For modeling, we solved numerically the system of coupled Poisson-Nernst-Planck-Navier-Stokes equations which governs transport of ion in the system. The finite volume method was employed for discretizing the equations. As the equations are nonlinear, we applied Newton method and GMRES in solving them^22^.

## Results and Discussion

### Current Utilization (CU) and Energy per Ion Removal (EPIR)

**Figure 1:**
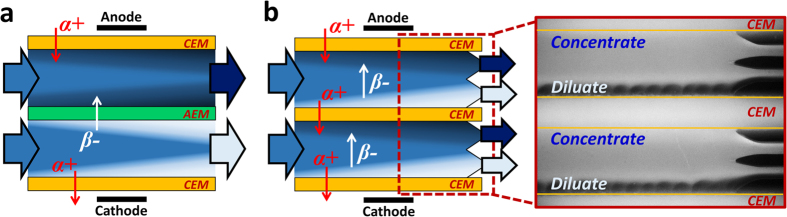
Schematic view of electrical membrane desalination. (**a**) Electrodialysis (ED) and (**b**) Ion Concentration Polarization (ICP) desalination with fluorescent image. Red and white arrows indicate the movement of cation *α*+ and anion *β*- by electric field, respectively. Bright and dark colored region in these schematic Figures represent ion depleted and enriched region, respectively while the fluorescent image shows an opposite contrast. ED utilizes both anion exchange membrane (AEM) and cation exchange membrane (CEM) for bipolar conduction while ICP desalination utilizes only CEMs for unipolar conduction. Movie file for standard ICP desalination is available in supplementary data.

**Figure 2:**
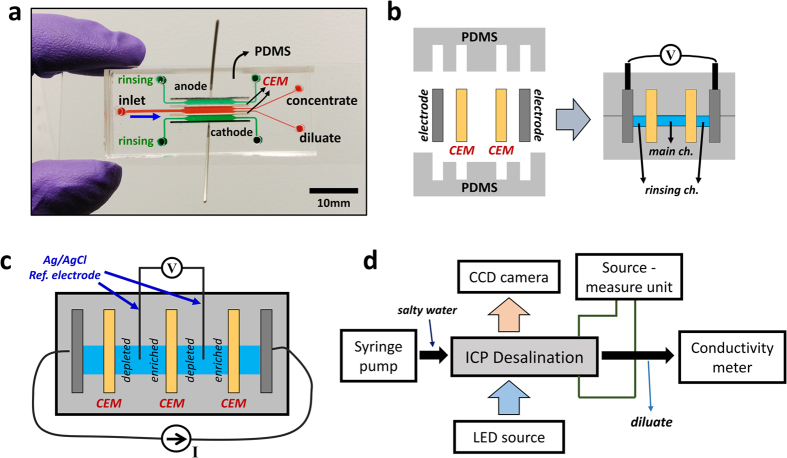
(**a**) Photo of PDMS-based ICP desalination device (**b**) Schematic view of cross-section (**c**) Measurement of unit cell voltage under constant current applied (**d**) Overall experimental setup.

In order to reveal general trends of important metrics of the desalination process, we experimentally characterized the ICP desalination under high salinity feedwater input at various operating current values. To investigate energy efficiency of ICP desalination in different feed salinity level, voltage drop (*V*) across the unit cell and concentration change (*C*_*0*_* − C*_*diluate*_) in dilute channel were measured under constant current applied to calculate two efficiency metrics of desalination – energy per ion removal (EPIR) and current utilization (CU),
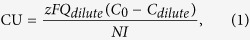

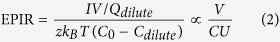
where *z, F, k*_B_*T* indicate ion valence, Faraday’s constant (= 9.65×10^4^ C·mol^−1^), and thermal energy (= 2.479 kJ/mol, *k*_B_ and *T* are Boltzmann constant and temperature) respectively. *V* is voltage, *I* is current, *N* is cell number of ED, *C*_*0*_ is initial ion concentration, *C*_*diluate*_ is ion concentration of dilute flow, and *Q*_*diluate*_ is flow rate of dilute flow. CU represents how efficiently current is used for a salt reduction in dilute flow. (Ideally it is unity in ED system if no current loss.) For fair comparison with conventional electrodialysis (a pair of one dilute and concentrate flow is repeated in parallel), we take two separate flows with three CEM as unit cell number (N = 1) as shown Fig. 1. By combining energy consumption (= *IV/Q*_*diluate*_) and salt removal ratio (= (*C*_*0*_ − *C*_*diluate*_)/*C*_*0*_), EPIR (*i.e.* specific energy) is calculated indicating how much energy is consumed to remove unit ion.

It has to be mentioned that ICP desalination with NaCl feed solution can achieve high CU value beyond one mainly because of diffusivity difference between cation and anion as our group already reported^16^. This can be explained by the three primary points; (i) depletion regions of AEM and CEM are independent, and (ii) CEMs, under the same current and with NaCl, generates stronger depletion zone than AEMs, mainly because of electroneutrality and current conservation met separately on each depletion regions, (iii) thus ICP desalination utilizing only CEMs can larger amount of salt removal in dilute flow, compared to the electrodialysis.

### Analysis of ICP desalination: Experiments and Numerical modeling

**Figure 3:**
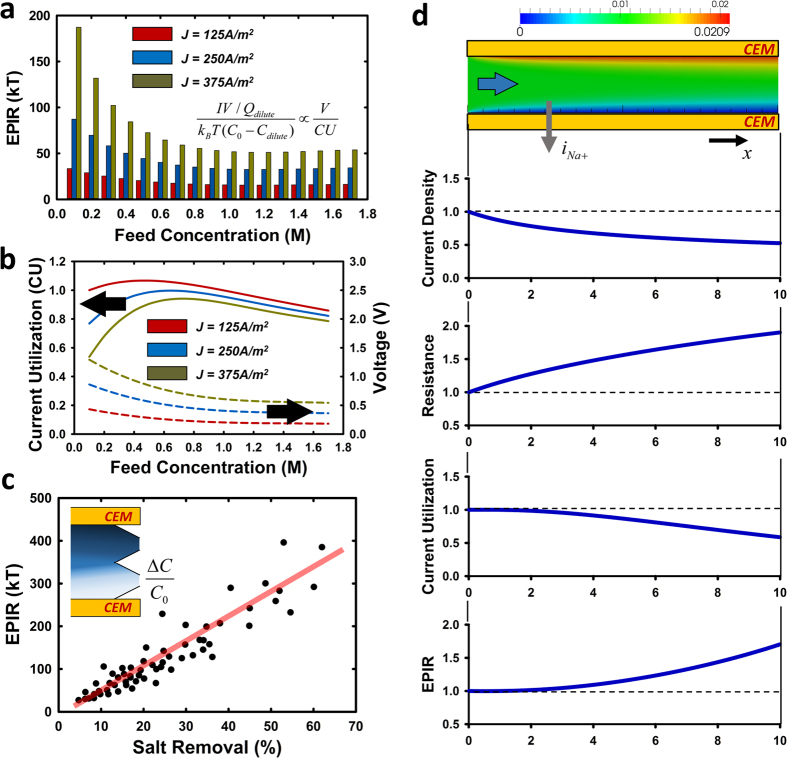
Evaluation of ICP desalination for experiments and numerical simulations. (**a**) Tendency of energy per ion removal (EPIR) and (**b**) current uilization (CU)/voltage depending on the feed concentration (0.1–1.7 M NaCl, equivalent to 5,800–100,000 mg/L of TDS) under constant current density. EPIR, expressed as a function of CU and voltage, rapidly decreases as feed concentration increases up to 0.8 M NaCl and afterwards keeps low value. The EPIR trend is more dominated by voltage decrement due to low feed and membrane resistance, rather than CU. (**c**) Linear relationship between EPIR and salt removal ratio under constant feed concentration (0.1 M NaCl) regardless of changing experimental parameters (*i.e.* current density, flow velocity, and membrane length). (**d**) Numerical simulation; Distribution of local current density, electrical resistance, CU and EPIR along the membrane expressed as dimensionless values. Increment of local electrical resistance due to growing ion depletion zone along the membrane results in not only poor CU and EPIR, but also deterioration of membrane area efficiency.

Figure 3a is the EPIR value calculated from the experimental results with various feed water salt concentrations. EPIR precipitously decreases with increased salinity below 0.8 M NaCl, before reaching a plateau. Figure 3b shows the voltage drop and CU changes, measured from experiments, depending on the feed concentration. This behavior could be explained from considering voltage drop across the membrane, as membrane itself becomes more conductive^23^ when higher feed concentration, but also lower electrical resistance in the main channel, with thinner ion depletion layer and more conducting ions present in feed water. CU typically decreases as feed concentration increases, mainly due to loss of membrane permselectivity. Poor CU observed in low salinity conditions may be due to the fact that the current applied is in the overlimiting regime, which causes undesirable chemical reactions such as water splitting^24^. Another evident trend is that lower operating current brings about lower EPIR in general, regardless of feed concentration and other parameters. In any operating condition, increasing the operating current will enlarge the size of depletion region, therefore increasing the cell resistance in general. Figure 3c shows experimental results of EPIR vs. salt removal ratio (effectively the size of depletion region) over a wide variety of parameter values (current density, flow velocity, and membrane length). Regardless of changing parameters, EPIR is always linearly proportional to the salt removal ratio, leading to the conclusion one achieve optimal energy efficiency (lowest EPIR) at the limit of incremental salt removal ratio, regardless of conditions.

In addition to the experimental results above, we also carried out multi-physics modeling of ICP desalination process we developed previously^22^, in order to investigate transport of salt ion in the device. The coupled Poisson-Nernst-Planck and Navier Stokes equations were solved directly used the solver. By adopting 1 mm of membrane length and 0.1 mm of the gap between the membranes to match up the ratio (1:10) with experiments, we numerically calculate local value of current density, electrical resistance, CU and EPIR to understand ion transport along the membrane. (Figure 3d) For an intuitive and relative comparison, numbers are expressed as dimensionless values divided by the local value in the beginning of the membrane. It is clearly observed that a majority of ion transport happens in the initial region of the membrane, where the ion depletion layer is thinner and thus, the local electrical resistance is lower. As ion depletion zone (*i.e.* electrical resistance) grows along the membrane, current flux through the membrane decreases mainly due to high electrical resistance, thereby resulting in poor CU and EPIR. In addition to the expected decline of energy efficiency, longer membrane should bring about reduced membrane area efficiency, closely related to the capital/maintenance cost. Considering the experimental and mathematical results above, one can consider less salt removal ratio (smaller driving current) and shorter membrane length as important direction for optimization, eventually driving us to come up with multi-stage (*i.e.* batch) operation in high salinity water treatment.

### Presenting multi-stage ICP desalination and optimization

**Figure 4:**
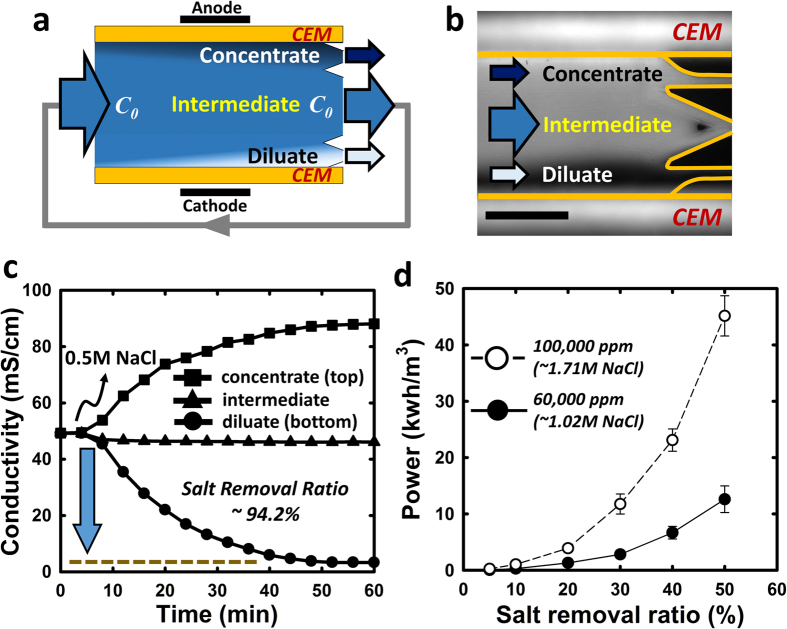
Trifurcated ICP desalination for multi-stage operation. (**a**) Schematic view of trifurcated ICP desalination. To obtain thin depletion stream and small amount of dilute flow with high purity, one can trifucate the main channel into three different output flows in accordance with concentration distribution between membranes. (SI Fig. 1a) Intermediate stream can be fed to the next stage by batch process or recirculation. (**b**) Fluorescent image of trifurcated ICP desalination using a feed solution of 0.5 M NaCl. Scale bar indicates 1 mm. (**c**) Real-time conductivity measurement of concentrate, intermediate, and dilute flows. Long saturation time is due to necessary volumetric capacity of micro conductivity meter (~17 μL), low dilute flow rate (~1.5 μL/min) and inevitable dead volume in fluidic connection. (**d**) Power consumption depending on different salt removal ratio for 60,000 ppm & 100,000 ppm feed water.

A key challenge to overcome in the multi-stage strategy is making thinner ion depletion layer (*i.e.* less salt removal) in unit stages, yet eventually removing a large amount of salts by repeating the single unit operation, keeping minimal EPIR values. In order to achieve output flow with high salt removal ratio but low EPIR per unit stage, we conceived trifurcated ICP strategy splitting the feed stream into three different output flows in accordance with concentration distribution between membranes (SI-section 1) shown in Fig. 4a. By the trifurcated ICP desalination, one can produce a small dilute flow with high purity and small concentrate flow with high impurity, while majority fluid in the middle can be delivered to the next stage as feed water either by batch or recirculation process. A detailed scheme for multi-staging trifurcated ICP process is shown in SI-section 2, where one can define overall recovery ratio as well as the ‘average number of stage’, which is dependent on the dilute production ratio of each stage operation. It has to be noted that each stage should be identical since our multi-stage operation aims to have volume-incremental output product and volume-decremental intermediate product under the constant operating conditions. (*e.g.* feed/product salinity, voltage/current) while multi-stage electrodialysis has different operating conditions in each stage^25^. To verify the practicality of the strategy extracting three distinguishing flow, trifurcated ICP desalination was operated and visualized using 0.5 M sodium chloride (NaCl) solution as shown in Fig. 4b. As a result, dilute stream shows high conductivity change up to 94% keeping the conductivity of intermediate stream almost constant as shown in Fig. 4c. One of the noticeable features of trifurcated ICP desalination is keeping feed concentration constant in every stage which enables us to play only in economically favorable EPIR regime (high salinity), not going through the extensive feed concentration changes between inlet and outlet channel in electrodialysis. In addition, there is less inter-channel concentration difference, which results in considerable voltage drop^26^, compared to the multi-stage electrodialysis as stage proceeds. Finally, to evaluate the power consumption with different feed salinities (60,000 ppm and 100,000 ppm), we operated the unit ICP desalination process (basis for multi-stage desalination system can be found in SI-Fig. 4) to achieve salt removal ratio of each 5, 10, 20, 30, 40, 50% by applying different current densities. (Figure 4d) Here, you can find that (1) the incremental rate of power consumption seems to be almost linear to the salt removal ratio, and (2) relative ratio of power consumption to desalinate 100,000 ppm and 60,000 ppm feed with same salt removal ratio can be observed to be larger than the salinity difference ratio. This is mainly due to the difference of current density since higher current density increase the electric resistance and decrease CU at the same time. So, in order to reduce the power consumption for certain salt removal ratio, one can readily come up with several methods to decrease the required current density such as lowering flow velocity, extending the membrane length, or reducing dilute flow rate. But, one should note that reduced power consumption does not always mean lower overall cost, so it is important to consider other cost factors in addition to power consumption. In this regard, we decided to change the dilute flow rate for certain salt removal ratio in order to examine the relationship between energy and cost.

**Figure 5:**
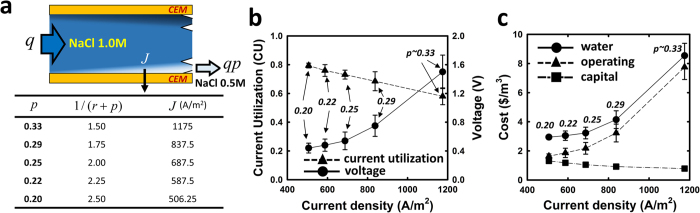
Strategy for optimizing multi-stage operation. (**a**) List of operation examples for different dilute production ratio (*p*) with average stage number (1/(*r* + *p*), and corresponding current densities for same salt removal ratio (50%) using a feed solution of 1 M NaCl. Definition of production ratio and average stage number is described in SI-section 2. (**b**) Tendency of CU and voltage depending on the applied current density (or production ratio). High current density reduces CU due to undesirable chemical reactions and back diffusion while augments potential drop across the unit cell due to low fluidic resistance and high membrane resistance. Since lower production ratio means more energy efficiency but also more stages required to deal with fixed feed volume quantity, multi-stage strategy should be optimized to achieve low (**c**) overall water cost. Feed flow rate (*q*) was fixed as 20 μL/min in all cases and analysis method of water cost is described in SI-section 3.2.1 & 3.2.2.

With a trifurcated ICP configuration, we experimentally characterized the electrical energy needed at different dilute production ratio (*p*) with the fixed amount of salt removal ratio achieved (50% salt removal ratio achieved with 1 M NaCl feedwater). In case of bifurcated ICP (~single stage desalination) as schematized in Fig. 1, *p* value is 0.5, which means 50% volume of dilute output and 50% volume of concentrated output without intermediate output volume. When the *p* value becomes smaller (than 0.5), more stages (or recirculation process) are needed to deal with certain volume of feed water. For several different production ratios, applied current density was regulated in each case by monitoring the conductivity of dilute output to provide the same (50%) salt removal ratio in the dilute output stream (Fig. 5a). As shown in Fig. 5b, in order to increase the production ratio processed, one needs higher current density, resulting in higher voltage drop, lower CU, and increased power consumption. Yet, lower production ratio means more average stage number are needed to process a given volume of brine feedwater, which results in higher capital cost that are dominated by the amount of membrane used^17^,^25^. Therefore, comparison between different production ratio conditions can produce total cost comparison ($/m^3^) among trifurcated multistage ICP brine desalination with different number of stages. We calculated the capital cost as a sum of membrane/equipment expenses and maintenance cost while operating cost was calculated solely by power consumption neglecting other minor costs, which was the similar methods used by other studies^17^,^27^,^28^. (details found in SI-section 3.2.1) Fig. 5c shows that reduced production ratio and current density lower the operating cost, while raising the capital cost (more average stage number/recirculation necessary to treat a given amount of feed water). Still, one can see that the overall cost for water processing (inclusive of both capital and energy cost) are optimized in the limit of more stages (incremental desalination at each stages). Further decrease in current, however, will bring about the limit where the capital cost overwhelms the energy cost, as was seen in the similar characterization using different NaCl concentration as the feed salinity. This is why we simply should not count on power consumption when we evaluate a certain technology since power consumption is just one of the major consideration, not the absolute metric itself for overall operation. (SI-section 3.2.2) In general, the optimal staging condition is expected to be dependent on the feedwater salinity, with saltier feedwater requiring more stages. (SI-section 3.2.3).

### Cost analysis

**Figure 6:**
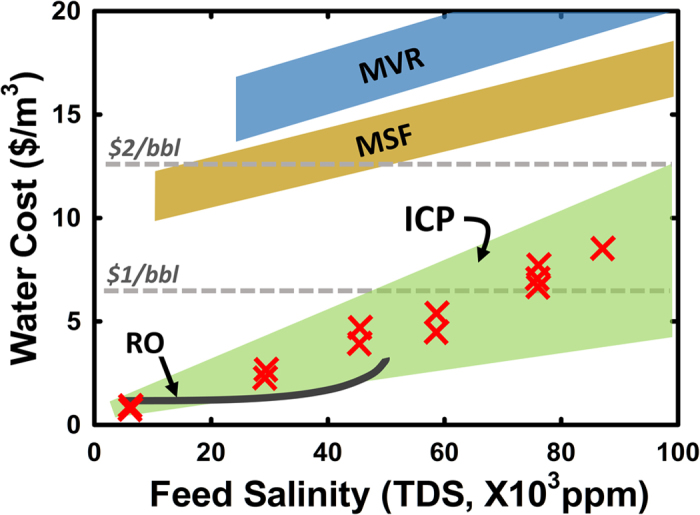
Cost analysis of ICP desalination. 12 red X denote results of ICP desalination based on the experiments with fixed recovery of 0.5 and 70–75% salt removal ratio from various feed salinity (5,800–87,700 mg/L of TDS, equivalent to 0.1–1.5 M NaCl solution), where most of flowback and some of produced water range. RO (not practically used beyond 45,000 mg/L of TDS) and MSF cost were calculted by open source program, *DEEP. Ver. 5*, shared in UN IAEA website. MVR cost was approximated from other reports^7^,^8^,^9^ and commercial factsheet. Pretreatment (mostly considered around $1/bbl), transportation, and labor cost were not considered here. Although the comparison shown here are based on several approximations and estimations (described in detail in the supplementary information), ICP cost (colored as green zone) compares favorably over other technologies such as MSF and MVR.

It has to be mentioned that the overall cost optimization carried out above is within several experimental constraints (Details are found in SI-section 3.2.4), therefore should not be considered as ultimate optimal cost for ICP desalination at given feed salinity. We carried out similar experimental characterization of ICP desalination power consumption on various feed salinity (5,800–87,700 mg/L of TDS, equivalent to 0.1–1.5 M NaCl solution), and estimated optimal overall cost of ICP desalination. (SI-section 3.2.4) The result is summarized in Fig. 6, where we also compared costs for other brine desalination technologies in the same plot. RO is not economically viable in brine desalination, because of rapid increase of power consumption and decrease of recovery ratio as feed salinity rises. In addition, RO generally require rigorous pretreatment and feed conditioning to prevent membrane fouling, the cost of which is typically larger than 1$/bbl^6^,^7^,^29^. Although MSF is widely used in seawater desalination, it requires significant infrastructure and equipments, which increases overall cost^30^. MVR is currently used for treating produced water from oil and gas industry, but the cost ranges $3.50–6.25/bbl ($22–39/m^3^) including capital cost, power consumption, labor, and other treatment expenses while sole operating cost is roughly ranges $2–3/bbl ($12.58~18.87/m^3^)^7^,^8^,^9^. This relatively high cost of brine desalination prevented (environmentally preferred) recycling of brine wastewater in oil and gas industry, unless forced by excessive disposal expenses (Details are plotted in SI-section 4), strict regulations, or high fresh water cost in shale or off-shore development. Moreover, all the above technologies produce only completely desalted water, which is often not necessary in many applications such as Enhanced Oil Recovery (EOR)^10^, where one can tolerate certain amount of salt in wastewater before recycling. According to our calculation, overall water cost of ICP desalination (treating the source water to 70% removal of the salt), is expected to be less than $1/bbl, for the source water up to 70,000 mg/L of TDS, with generally linear dependence of overall cost on the feed water salinity. As conventional water treatment process encounters more and tougher challenges^31^, such a flexible, partial desalination^11^ is potentially the most cost-effective option for economically viable water reuse in oil and gas industry as well as brine management, since partially desalted output streams can be effectively treated by other technologies or recycled for other applications.

While this cost estimation for multi-stage ICP desalination is based on the small scale experimental model and therefore preliminary, it certainly warrants further scale-up engineering efforts to realize this projected gains in energy and cost efficiency in brine desalination. We used a relatively high-resistance membrane (Fumatech FTCM-E, area resistance ~13.3 Ω · *cm*^2^) for our microfluidic characterization of the brine desalination process. Therefore there is much room for further improvement, because power consumption for desalination is strongly dependent on the membrane resistance in electrical desalination system^24^. In addition to trifurcated ICP strategy, there are other possible multi-stage strategies tailored to specific purpose. (SI-section 5) System recovery, fixed as 50% here for simplicity of comparison, could be enhanced further, taking into account asymmetric concentration distribution between membranes. (SI-Fig. 1a).

### Removal of suspended solids

**Figure 7:**
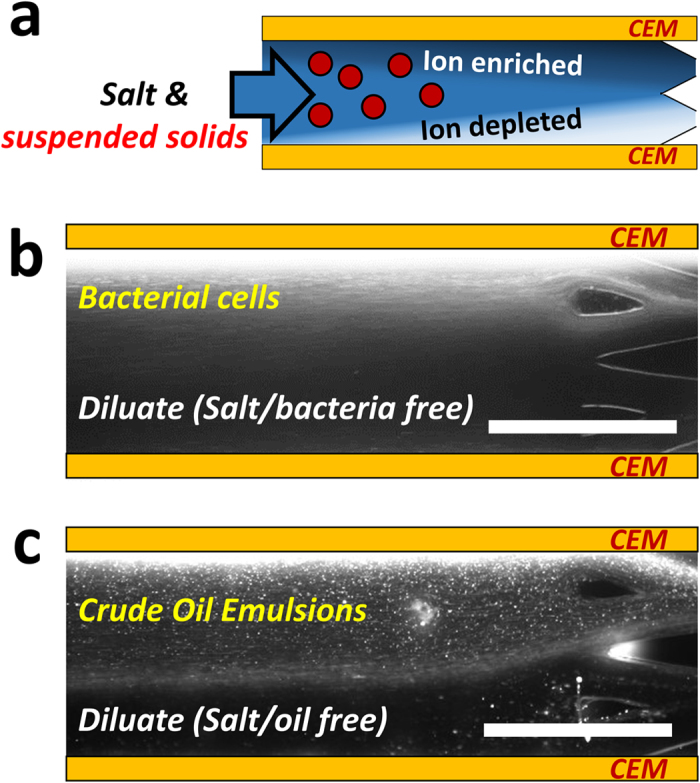
Demonstration of simultaneous reduction of total dissolved solids (TDS) and total suspended solids (TSS). (**a**) Schematic illustration of suspended solids removal along with salts. Negatively charged particles en bloc could be delivered to the concentrate stream on top by electromigration and electroconvection while salt ions removed through the membrane on bottom. Demonstration of suspended solids removal out of dilute stream for (**b**) Bacterial cells (*Escherichia coli*) and (**c**) crude oil emulsions. Details are described in method section and all scale bars indicate 1 mm. A movie for crude oil emulsions are available in supplementary data.

Since brine wastewater (*e.g.* produced water) typically contains a great deal of suspended solids as well as salts, most desalination technologies require several pretreatment steps (*e.g.* settling/sedimentation and filtration) to reduce Total Suspended Solids (TSS), which complicate treatment processes and increase the overall water cost^32^. Even after pretreatment, remaining TSS can accumulate on plumbing or membrane surface, eventually causing operational challenges. We present the capability of ICP desalination to remove most of charged suspended solids while keeping removing salts through the membrane at the same time. Previously, our group demonstrated microfluidic continuous biomolecule concentration near CEM (*i.e.* Nafion membrane)^33^, but ICP desalination presented here is readily scalable in the same manner as electrodialysis. Once negatively charged particles dispersed in the feed flow are introduced in the fluidic channel as shown in Fig. 7a, they can be delivered to the concentrate stream on the top mainly by an electrophoretic force on the charged species. Although this delivery of charged particles *en bloc* can also happen in the electrodialysis, they would not be streamed out from the dilute output flow in the absence of separation of output flow. In order to observe the removal capability of charged particles, *Escherichia coli (E. coli*) and crude oil emulsions, which are of main concerns in desalination due to fouling, were added in feedwater as shown in Fig. 7b,c, respectively. As shown in the fluorescent images, one can observe that charged species linearly migrate upward (to the anode direction) and finally streamed out to the concentrate output flow. Since the majority of bio-contaminants (*e.g.* bacteria) and colloidal particles (*e.g.* oil emulsion) found in nature are of negative zeta potentials^34^, the ability to simultaneously remove both salts and various suspended contaminants is the one of the key distinguishing features in ICP desalination, potentially leading to the reduction of overall cost associated with pretreatment. However, a certain situation should be considered that colloidal particles can be aggregated and deposited on the membrane interface resulted from high interfacial interaction. We provided details for the interfacial interaction of colloidal particles and corresponding method to mitigate membrane issues. (SI-section 6).

### Membrane fouling and scaling

**Figure 8:**
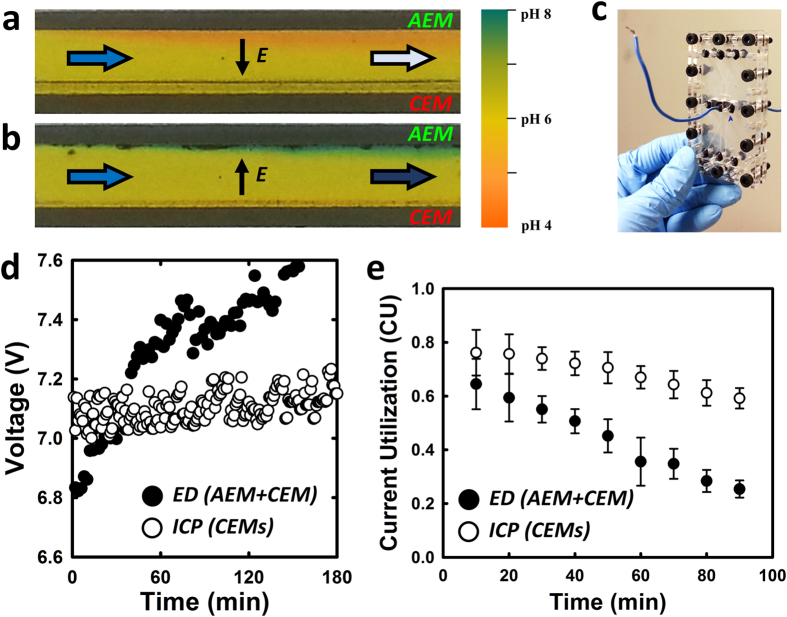
Verification for durability and stability of ICP operation. Observation of pH change adjacent to the AEM and CEM which is considered to be immediate indicator for salt scaling on the membrane. With increment of current from limiting current density, pH changes were always found near the AEM earlier than CEM where (**a**) acidification in dilute stream and (**b**) basification in concentrate stream in electrodialysis platform. Current density of 200 A/m^2^ was applied to the feed solution of 0.1 M NaCl and pH indicator was used for visualization. More results are presented in SI-section 6. To verify less succeptibility to membrane scaling/fouling, (**c**) plastic-based three channel stacked electrodialysis and ICP devices were prepared. (**d**) Measurement of electrical resistance change for three hours under constant current density of 500 A/m^2^ using natural seawater without any prefiltration or chemical precipitation and it shows better operating stability and durability against membrane degradation. (**e**) CU reduction ratio of electrodialysis is higher than that of ICP desalination.

Membrane-based desalination technologies all suffer from membrane fouling (accumulation of biomass)/scaling (accumulation of calcium deposits), which requires fastidious maintenance and replacement. Although it is generally known that ion exchange membrane is more robust and stable than RO membrane^35^, it is still challenging to minimize fouling/scaling on the membrane surface. Negatively charged organic foulants (especially natural organic matter with a larger molecular weight) in many effluent and seawater foul AEMs up more significantly than CEMs. increasing the resistance of the membrane and inhibiting ion transport^35^,^36^,^37^. Scale formation (calcium deposits) in electrodialysis is also another challenge, originating from the pH shift adjacent to the membrane^35^, caused by water splitting (producing H^+^ and OH^−^), and often exacerbated by high current density, (*i.e.* overlimiting current regime). High pH values at the enriched interface of membrane render salt deposition on the membrane and it is mostly observed near AEM rather than CEM experimentally^38^. These membrane degradation processes lead to a significant increase of the membrane resistance and retarding counter-ion transport, thereby requiring numerous remedies such as electrodialysis reversal (EDR), pulsed current operation, chemical additives for pH adjustment, and pretreatment^38^,^39^,^40^. In this regard, deterioration of membrane performance in ICP desalination is expected to be much less than the electrodialysis, simply because ICP process can be operated without AEMs. From the microfluidic device operation, we could monitor any pH change adjacent to the each AEM and CEM by adding pH indicator dyes into the feed water. As shown in Fig. 8a,b, (Details are available in SI-section 7) it is clearly observed that significant pH changes were observed near the AEM, but not CEM, and also some of the particle aggregation were easily found near AEM only. Followed by pH visualization, we monitored the voltage drop (for tracing the membrane resistance) and CU changes over time by running three channel stacked electrodialysis and ICP unit (Fig. 8c) under constant current density (*J* = 500 A/m^2^) applied, with a feed of natural seawater (Revere beach, MA) without any pre-filtration step. As shown in Fig. 8d, it is found that initial voltage of electrodialysis is less than voltage of ICP system mainly due to intrinsic difference of area resistance between AEM and CEM by vendor. However, voltage drop of electrodialysis continues to increase over time, probably due to membrane fouling by diverse organic foulants in natural seawater, while ICP voltage remains largely constant over an extended period of time, demonstrating much better long-term stability of desalination operation. In addition to the voltage measurement, we also calculated CU of each system based on the conductivity measurement of dilute output flow for 90 minutes. Figure 8e represents that CU reduction ratio of electrodialysis is higher than that of ICP desalination which could be well explained by the fact that more fouling on the membrane surface disturb counter-ion transport through the membrane.

### Removal of divalent cations

In many industrial water treatment applications, removal of divalent cations is considered to be as important as the reduction of TDS and TSS. For example, divalent cations should be removed in recycling treatment of flowback or produced water, since they form carbonate or sulfate precipitates in the wellbore, leading to scaling and reduction in production^6^,^8^. Divalent cations would be proportionally removed more or equal to monovalent cations at least in electrodialysis^41^,^42^,^43^, which is the same feature for ICP desalination in our testing (SI-section 8) with sodium and calcium cation.

## Conclusion

In this work, we have demonstrated ICP desalination for treating ultra-high salinity water treatment (up to 100,000 mg/L of TDS) by presenting multi-stage operation based on the analysis of energy and water cost. Additionally, we have shown the simultaneous removal of suspended solids (crude oil emulsions & bacteria) and salts. Lastly, we have identified that ICP desalination is less susceptible from scaling/membrane fouling, which is a significant challenge in any membrane-based technologies. Unique advantages of ICP described in this work could address many challenges faced in brine treatment associated with most of the oilfield development.

## Additional Information

**How to cite this article**: Kim, B. *et al*. Purification of High Salinity Brine by Multi-Stage Ion Concentration Polarization Desalination. *Sci. Rep.* **6**, 31850; doi: 10.1038/srep31850 (2016).

## Supplementary Material

Supplementary Information

Supplementary Movie 1

Supplementary Movie 2

Supplementary Movie 3
