# Corrigendum: Purification of High Salinity Brine by Multi-Stage Ion Concentration Polarization Desalination

**DOI:** 10.1038/srep33775

**Published:** 2016-09-21

**Authors:** Bumjoo Kim, Rhokyun Kwak, Hyukjin J. Kwon, Van Sang Pham, Minseok Kim, Bader Al-Anzi, Geunbae Lim, Jongyoon Han

Scientific Reports 6: Article number: 3185010.1038/srep31850; published online: 08 22 2016; updated: 09 21 2016

This Article contains errors in Reference 19.

“Kwak, R., Pham, V. S., Kim, B., Chen, L. & Han, J. Enhanced current utilization by unipolar ion conduction in ion concentration polarization desalination. *submitted* (2016)”.

should read:

“Kwak, R. *et al.* Enhanced salt removal by unipolar ion conduction in ion concentration polarization desalination. *Sci. Rep.* **6**, 25349; doi: 10.1038/srep25349 (2016)”.

