# Corrigendum: Purification of High Salinity Brine by Multi-Stage Ion Concentration Polarization Desalination

**DOI:** 10.1038/srep46918

**Published:** 2018-06-08

**Authors:** Bumjoo Kim, Rhokyun Kwak, Hyukjin J. Kwon, Van Sang Pham, Minseok Kim, Bader Al-Anzi, Geunbae Lim, Jongyoon Han

Scientific Reports 6: Article number: 3185010.1038/srep31850; published online: 08 22 2016; updated: 06 08 2018

In this Article, Van Sang Pham is incorrectly affiliated with ‘Singapore-MIT Alliance for Research and Technology (SMART) Centre, Singapore’. The correct affiliations for Van Sang Pham are listed below:

Department of Electrical Engineering and Computer Science, Massachusetts Institute of Technology, 77 Massachusetts Avenue, Cambridge, MA 02139, USA.

Hanoi University of Science and Technology, Vietnam.

