# Supplementary material for: Purification of High Salinity Brine by Multi-Stage Ion Concentration Polarization Desalination
Source: Sci Rep. 2016 Aug 22;6:31850. doi: 10.1038/srep31850 (PMC4992824; doi:10.1038/srep31850)
Supplement: Supplementary Information [file srep31850-s1.pdf]

Supplementary Information for

Purification of High Salinity Brine by Multi-Stage Ion Concentration Polarization Desalination

Bumjoo Kim, Rhokyun Kwak, Hyukjin J. Kwon, Van Sang Pham, Minseok Kim,

Bader Al-Anzi, Geunbae Lim, and Jongyoon Han†

†To whom correspondence should be addressed. E-mail: jyhan@mit.edu

1. Definition of current regime and voltage-current response

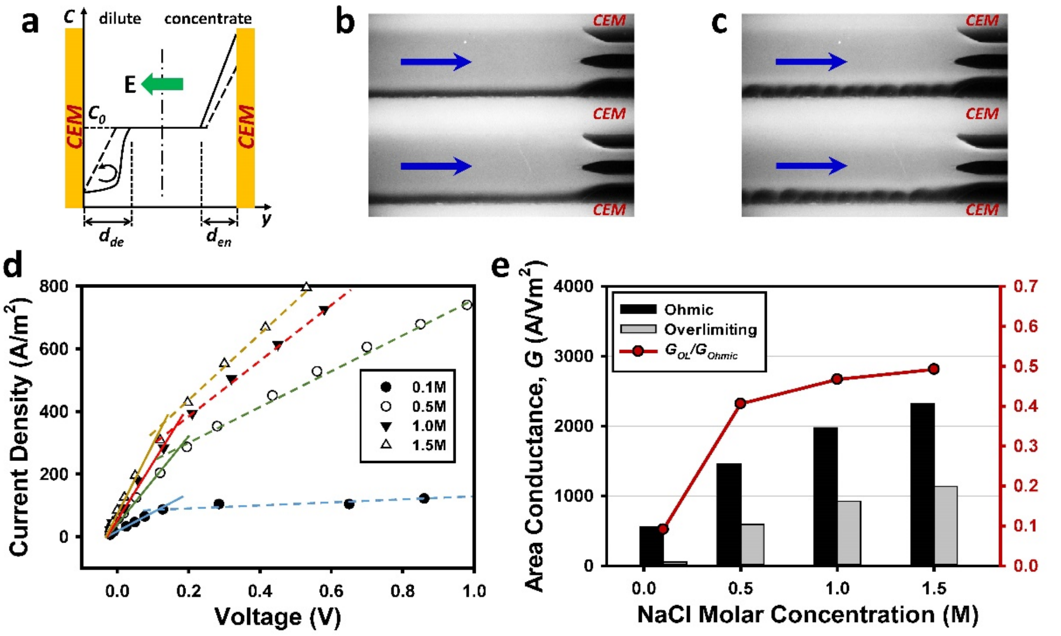

SI-Fig. 1. (a) Concentration distribution between CEMs. Ion depletion zone d_{ec} with low ion concentration and Ion enrichment zone d_{en} with high ion concentration. Dotted and solid line represent Ohmic and overlimiting regime, respectively. Fluorescent images of (b) Ohmic and (c)

overlimiting desalination. (d) Voltage-current response for feed solution of 0.1-1.5M NaCl. (e) Comparison of cell conductivity on Ohmic and overlimiting regime in each concentration.

Generally, the limiting current density $i_{\lim}$ (A/m^2) depends on the bulk solution concentration C_0 (mol/L) and expressed as

$$i_{\lim} = \frac{F \cdot C_0 \cdot D}{\delta(t^m - t^s)},$$

where D (m^2/s) is a salt diffusion coefficient, δ (m) is boundary layer thickness, F (A·s/mol) is the Faraday constant, and t^m, t^s are counterion transport number in membrane and solution, respectively. The limiting current is often considered as the upper limit of optimized operating conditions for electrochemical systems with IEMs, and overlimiting operation is often considered energetically inefficient. However, desalination in the overlimiting regime needs to be re-evaluated especially in the light of minimizing the capital cost (reducing the area of expensive IEMs necessary to achieve the required level of desalination).[1] In our studies, current density ranges from limiting to early overlimiting regime represent optimal operating conditions, especially for higher salinity feed conditions. One critical feature of electrical desalination in overlimiting current regime is that current-voltage response shows that cell resistance in overlimiting regime is almost comparable to one in Ohmic regime in high bulk concentration (> 0.5M). (SI-Fig. 1d and 1e) An overlimiting operation at lower salinity (0.1M, filled circle in SI-Fig. 1d) would have posed significantly higher voltage drop (energy consumption), cell resistance is only modestly increased in the overlimiting regime in high salinity conditions. Therefore,

overlimiting operation can represent optimal (in terms of overall cost, inclusive of energy and capital cost) operating condition if feedwater salinity is significantly high.

2. Multi-staging scheme for trifurcated ICP desalination

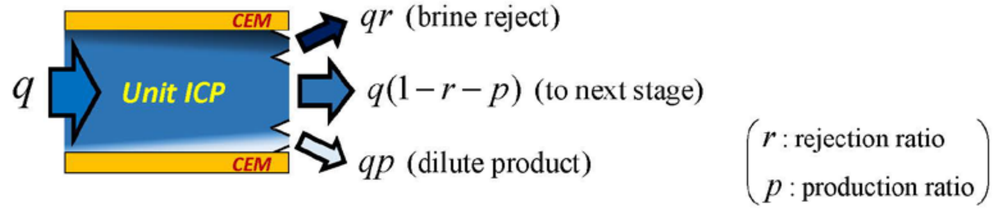

p is determined by the optimization.

r can be different, but for simplicity, we set $r = p$ here. (overall recovery ~ 0.5)

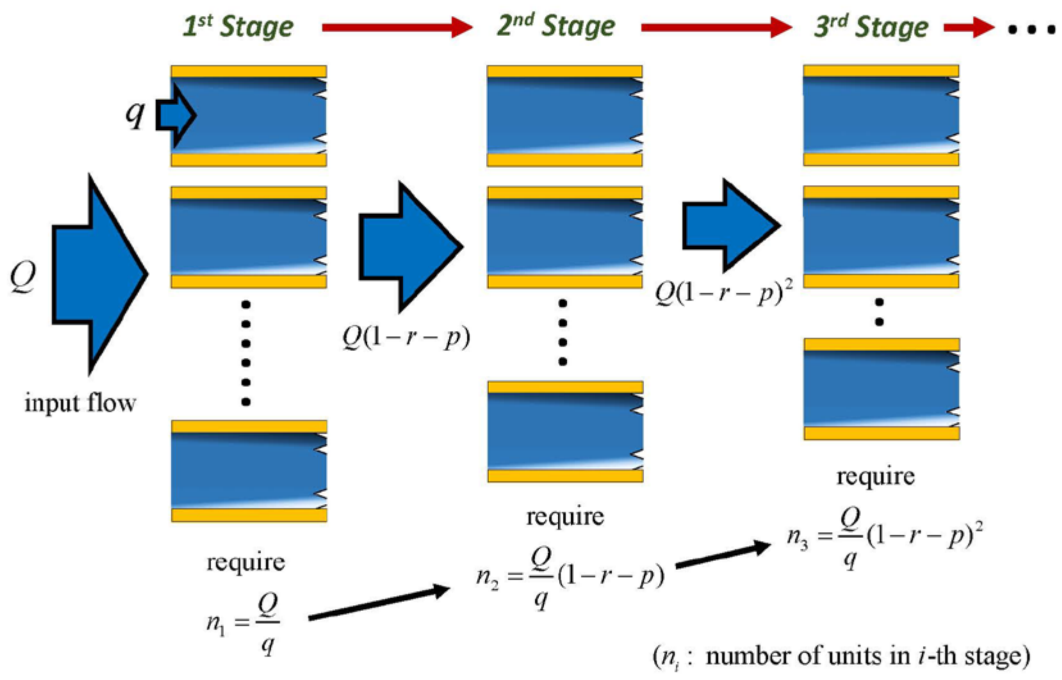

Total number of individual unit ICP necessary to process Q

$$N_{total} = n_1 + n_2 + \cdots = \frac{Q}{q}\left\{1 + (1-r-p) + (1-r-p)^2 + \cdots\right\} = \frac{Q}{q}\cdot\frac{1}{r+p}$$

when $r = p = \frac{1}{2}$ $\Rightarrow$ single stage operation (high energy cost)

$r = p = \frac{1}{4}$ $\Rightarrow$ "2" stage operation (on average, 2 stages required)

One can define $\frac{1}{r+p}$ as "average stage number"

SI-Fig. 2. Multi staging schemes for Trifurcated ICP desalination: One can define the unit system for trifurcated ICP desalination process where an input feedwater stream q can be processed to generate a diluted stream qp (with the production ratio p), a brine reject stream qr (rejection ratio r), with the remaining flow stream goes to the next stage for further processing. Both p and r can be adjusted freely by adjusting the flow split at the end of the unit device. For optimal cost efficiency, p should be optimized depending on input feedwater salinity as well as the salt removal ratio required (see SI-Fig. 4). r can be adjusted to achieve higher or lower overall recovery, but in our calculation we set $r=p$, which yields 50% overall recovery ratio (overall recovery ratio=$p/(r+p)$). Also, setting $r=p$ means the input stream salinity is maintained in all the stages (no further concentration of the feedwater in each stage), which is an optimal point of operation. Based on this, one can calculate the total number of unit system required to process a fixed input feedwater stream, as above.

In order to realize multi-stage operation, one could simply 1) connect many units (with the same process parameters) in series to treat the entire feed water volume (See SI-Fig. 3a below), or 2) recirculate the output feed back into the original feed flow, while maintaining smaller single unit processing rate. (In this case, we would need many units operating in parallel to process the same amount of feedwater (See SI-Fig. 3b below). As one can see below, in both scenarios the unit operation is exactly the same (same feed salinity, same salt removal ratio, and the same recovery).

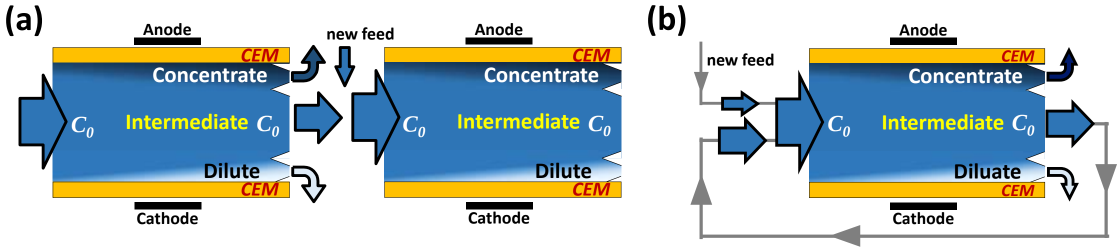

SI-Fig. 3. Schematics of multi-stage operation in ICP desalination; (a) multiple units connected in series, and (b) single unit for recirculation

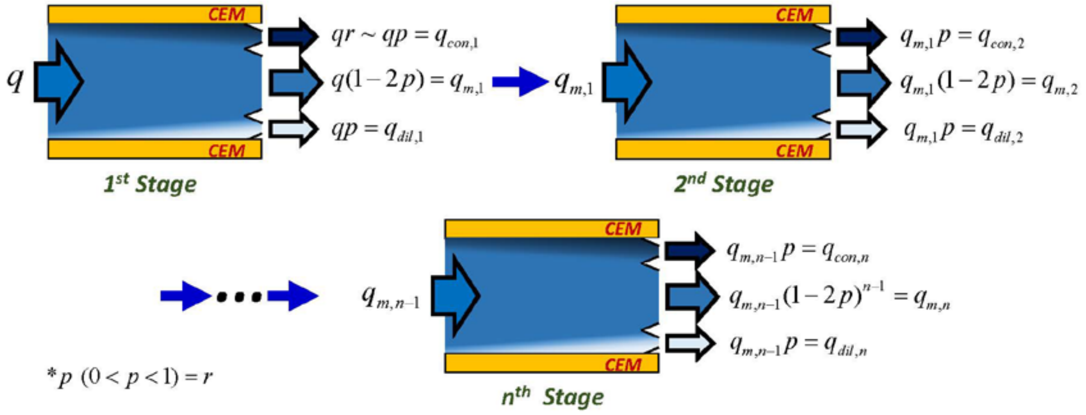

$$\text{Overall Recovery} = \frac{q_{dil,1} + q_{dil,2} + \ldots + q_{dil,n}}{q} = \frac{1}{q}\left[qp + qp(1-2p^2) + qp(1-2p^3) + \cdots + qp(1-2p^{n-1}) \right]$$

$$(0 < 1-2p < 1,\ n \to \infty) \qquad = \frac{1}{q} \cdot \frac{qp}{1-(1-2p)} = \frac{1}{2}$$

SI-Fig. 4. Calculation of overall recovery showing that same flow rate in dilute and concentrate stream in unit stage results in overall recovery of 50%. We can assume trifurcated ICP would be operated as batch mode (not continuous mode in ED), which use single-stage unit and recirculate each dilute and concentrate stream for multi-stage operation. Here, in ICP batch mode, intermediate stream is recirculated while each dilute & concentrate stream should be extracted continuously. Based on our calculation, recovery depends on the flow rate ratio between dilute & concentrate output, regardless of intermediate flow rate.

3. Water Cost Analysis

3.1. Pumping energy

We simply calculated pressure drop by Hagen-Poiseuille (H-P) equation, the power consumption per stage, W can be expressed as

$$W = \Delta p \times Q = \frac{12\mu QL}{wd^3} \times Q$$
$$= \frac{12 \times 0.00972 g / cm \cdot s \times 20\mu L / \min \times 0.08m}{0.0018m \times (0.0002m)^3} \times 20\mu L / \min = 7.2 \times 10^{-9} (W)$$

where

μ : dynamic viscosity* $(N \cdot s / m^2 = kg / (m \cdot s))$	w : *width* $(0.0018m)$
Δp : pressure $(kg / m \cdot s^2)$	d : *depth* $(0.0002m)$
W : power consumption (Wh)	L : *length* $(0.08m)$
Q : *flowrate* $(20\mu L / \min)$	

Thus, power efficiency for pumping (per each stage) is

$$W / Q = \frac{7.2 \times 10^{-9} W}{20\mu L / \min} = 6mW / m^3$$

Based on the values obtained from the above calculation, we neglected pumping power for the calculation of water cost.

* Dynamic viscosity of salty waters (*e.g.* 1.0M NaCl solution) from J. Kestin *et al*, *J. Phys. Chem.*, 1981.

3.2. Water Cost

3.2.1. Calculation method

We calculated water cost as a sum of capital and operating cost. Capital cost includes cost for equipment (fluidic equipment + membrane) and maintenance cost while operating cost includes mainly power consumption. We excluded other minor cost such as pretreatment, replacement, transportation, and land, which highly depend on feed water condition and local situation. In detail, equipment cost can be expressed as

$$Equipment\ Cost\ (\$/m^3) = \frac{Used\ Membrane\ Cost\ (\$)}{Output\ Flow\ Volume\ per\ Life\ (m^3)} \times Annualised\ Factor = \frac{A_m \times K_Q}{Q_d \times T} \cdot \frac{(1+R)^T - 1}{T \times R}$$

and operating cost can be expressed as

$$Operating\ Cost\ (\$/m^3) = Power\ (KWh/m^3) \times Electricity\ Rate\ (\$/KWh) = \frac{V \times I}{Q_d} \cdot K_E$$

where A_m, K_Q, and Q_d indicate membrane area (m^2), area normalized equipment price ($\$/m^2$ membrane), and dilute flow rate (m^3/h), respectively. We assumed equipment costs (including membrane cost and corresponding other equipment cost) to scale with membrane area by applying the factor K_Q=\$1500/m^2 from previous literatures.[2-4] Unlike other cost, equipment cost is considered as up-front investment, lifespan (*T*, years) and annual interest rate (*R*, %) should be involved here. (*T*=20, *R*=0.06) *V* is voltage (V), *I* is current (A), and K_E is electricity rate (\$/KWh) set to \$0.11/KWh based on the average commercial electricity rate in 2014 from U.S. Energy Information Administration.[5]

Maintenance cost was roughly approximated as 25% of equipment cost.[6] The later term (=annualized factor) in equipment cost should be calculated as dimensionless term.

3.2.2. Water cost plot

Based on the calculation method described above, one can express water cost including the capital and operating costs as a function of operating current density of ICP desalination. As higher current density is applied in electrochemical desalination system (*e.g.* electrodialysis, ICP desalination), the operating (energy) cost increases due to an increase in electrical power consumption while the capital cost decreases due to a decrease in required equipment size (expressed in terms of membrane area required). As shown in SI-Fig. 5, the optimal water cost is determined by the combination of the operating and capital costs, where the operating and capital costs are comparable. However, this will also be affected by various cost factors, such as membrane unit cost, equipment cost, electricity, interest rate, maintenance cost, and others for different feed salinity.[1] In order to demonstrate the relationship between optimal staging condition and feed salinity, we examined water cost plots for different feedwater salinity. (NaCl 0.1M, 0.5M, and 0.8M) For the purpose of fair comparison to get the same salt removal ratio (~50%), we modulated the applied current for each different dilute production ratio (*p*, 0.18-0.33) by monitoring conductivity value in the dilute output. As shown in SI-Fig. 5, dilute production ratio of optimal cost point would be shifted to be lower values for higher feed salinity while optimal current density increases. In higher salinity, operating cost (energy cost) would increase rapidly, becoming dominant cost factor, so reducing energy cost (by decreasing *p* and increasing the number of stages) should be key to cost-

optimized desalination. These results, therefore, confirm the economic justification of multi-stage operation for treating high salinity brine water using ICP desalination process.

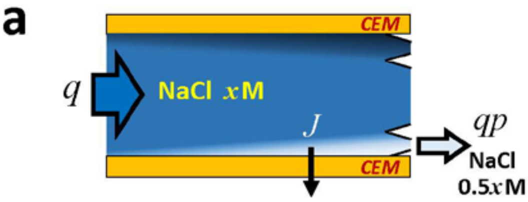

p	$1/(r+p)$	J (A/m²) 0.1M	0.5M	0.8M
**0.33**	1.50	195	630	813
**0.29**	1.75	162	520	644
**0.25**	2.00	138	440	531
**0.22**	2.25	120	380	456
**0.20**	2.50	105	340	400
**0.18**	2.75	93	310	350

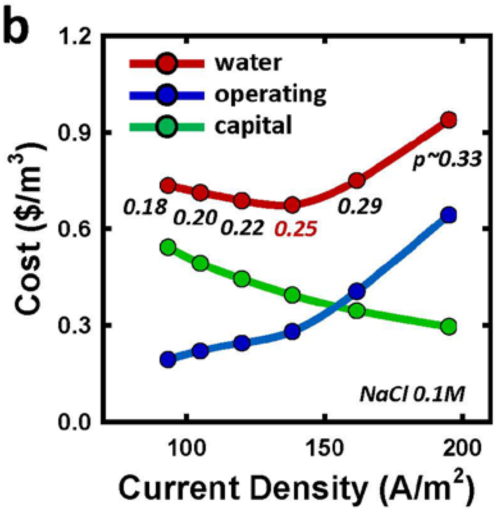

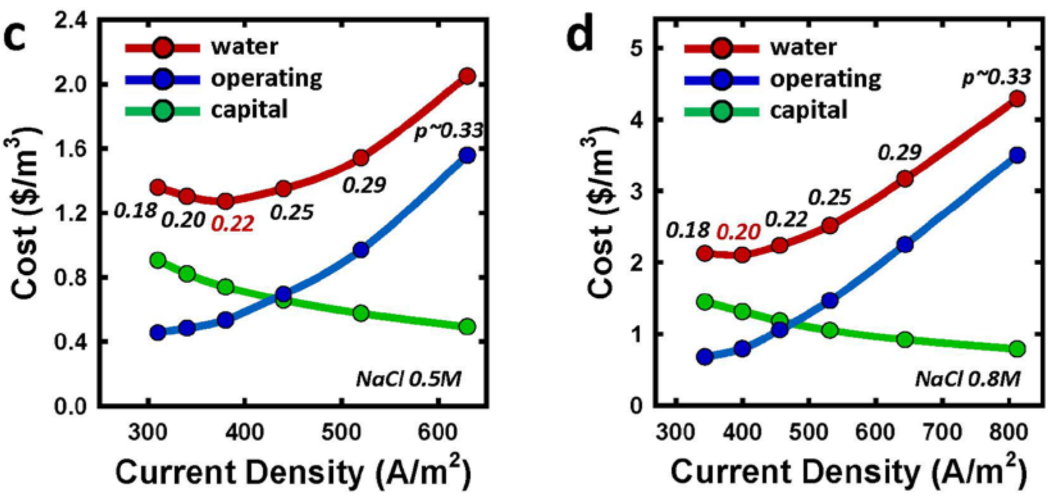

SI-Fig. 5. (a) List of operation examples for different dilute production ratio, average stage number, and current densities under same salt removal ratio (50%) using a feed solution of 0.1M/0.5M/0.8M NaCl feed solution. Corresponding water cost plot for (b) 0.1M, (c) 0.5M, and (d) 0.8M NaCl. Different dilute flow rate was applied to examine the optimal production ratio (colored in red) with average stage number, and one can clearly find out how many stages are necessary to treat feedwater for different salinity on average. Feed flow rate (q) was fixed as 20 μL/min in all cases.

3.2.3. Cost analysis (Figure 6)

We prepared multiple devices (200μm depth) with different channel length (30~100 ㎜) for different feed concentrations (NaCl 0.1~1.5M) since larger membrane area would be required as feed salinity increases under constant dilute flow rate (4μL). For each feed salinity, we modulated the applied current in order to get 70% of salt removal at least, and measured the corresponding voltage at the same time. Using the same method described above, we calculated capital cost from the applied flow rate & used membrane area, and operating cost from the applied current & measured voltage.

In respect of current technologies in produced water market, RO is not economically valid because of not only rapid increase of power consumption and decrease of recovery but also considerable pretreatment cost and maintenance (>$1/bbl) preventing membrane fouling.[7,8] MSF is widely used in seawater desalination mostly constructed in early stage before appearance of RO, but is rarely used for produced water treatment. MVR is governing technology for recycle of produced water and the cost ranges $3.50-6.25/bbl ($22-39/m^3) including capital cost, power consumption, labor, and other treatment while sole operating cost is roughly ranges between $2~3/bbl ($12.58~18.87/m^3).[8-10]

As mentioned in the manuscript, ICP water costs in Fig. 6 can be assumed as not fully optimized results, but estimation of expected ICP water cost domain mainly due to technical limit of fabricating microfluidic model ICP system for characterization. Since we examined ICP desalination based on the microfluidic platform with the aid of 3D

printer, our ability to test longer membrane channel (>100 mm), and smaller channel features for extremely small dilute/concentrate stream flow rate (in other words, operation at smaller *p(=r)* values). This means that the optimization of the cost here is not at all final, and can be further improved by fully expanding the design parameter (such as smaller *p* and Q_d values, which are important for higher salinity feedwater conditions). Still, the lowest cost demonstrated in our calculation based on experimentally achievable conditions in our study is still around 1$/bbl (which is often considered economically attractive price point for produced water treatment), so one can make a strong case for ICP desalination for brine desalination against other available technologies, especially when ICP systems are properly scaled up based on the optimization described here. On the other hand, 3D printed microfluidic ICP platform will be important in determining key design parameters of such scaled up systems, via visualization of ion transport phenomena / concentration mapping near commercialized IEM product, and direct measurement of effective potential drop (*i.e.* energy consumption) across the channels.

Basic Information			Calculation of Capital Cost			Calculation of Operating Cost				Water Cost ①+②+③	
Feed Conc.	Flow rate, Q_D	Membrane Area, A_m	Equipment ①	Maintenance ②	Overall Capital Cost ①+②	I	V	Power Cons.	Overall Operating Cost ③		
(mg/L)	(ul/min)	(m^2)	($/m^3)	($/m^3)	($/m^3)	(mA)	(volt)	(KWh/m^3)	($/m^3)	($/m^3)	($/bbl)
5800	4	0.000006	0.39	0.10	0.49	0.95	1.04	4.12	0.45	0.94	0.15
5800	4	0.000006	0.39	0.10	0.49	0.94	1.15	4.50	0.50	0.99	0.16
29200	4	0.00001	0.66	0.16	0.82	5.5	0.61	13.98	1.54	2.36	0.37
29200	4	0.00001	0.66	0.16	0.82	5.7	0.65	15.44	1.70	2.52	0.40
46800	4	0.000016	1.05	0.26	1.31	10	0.71	31.67	3.48	4.80	0.76
46800	4	0.000016	1.05	0.26	1.31	9.2	0.63	24.15	2.66	3.97	0.63
58500	4	0.000016	1.05	0.26	1.31	12	0.63	29.00	3.19	4.50	0.72
58500	4	0.000016	1.05	0.26	1.31	13	0.67	36.29	3.99	5.30	0.84
76000	4	0.00002	1.31	0.33	1.64	17	0.73	49.58	5.45	7.09	1.13
76000	4	0.00002	1.31	0.33	1.64	16.8	0.66	46.20	5.08	6.72	1.07
76000	4	0.00002	1.31	0.33	1.64	18	0.74	55.50	6.11	7.75	1.23
87700	4	0.00002	1.31	0.33	1.64	19.8	0.76	62.70	6.90	8.54	1.36

$$\text{Equipment Cost }(\$/m^3) = \frac{A_m \times K_Q}{Q_d \times T} \cdot \frac{(1+R)^T - 1}{T \times R}$$

$$\text{Maintenance Cost }(\$/m^3) \approx 0.25 \times \text{Equipment Cost}$$

$$\text{Operating Cost }(\$/m^3) = \frac{V \times I}{Q_d} \cdot K_E$$

$$K_Q = 1500(\$/m^2),\ T = 20(yr),\ R = 0.06(/yr),\ K_E = 0.11(\$/KWh)$$

Table SI-1. Summarized experimental information and calculation of ICP desalination for cost analysis indicated as red X in Figure 6 in the manuscript.

4. Evaluation of disposal cost in major oilfields in United States

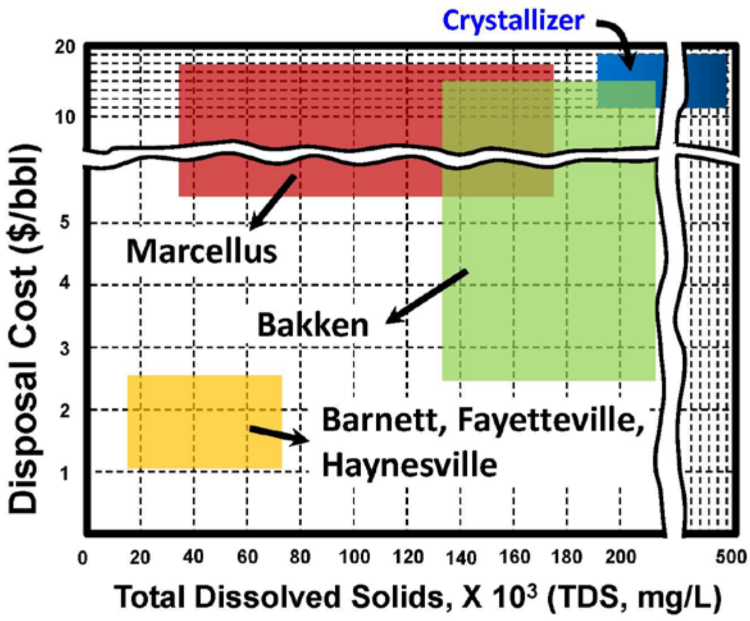

SI-Fig. 6. Current state of disposal cost in major shale development showing rough summary of cost and TDS range. Crystallizer is regarded as brine disposal treatment here.

Although there are slight difference about disposal cost values in many reports,[11-14] we summarized the waste water (produced water) disposal cost in order to overview of current state. (SI-Fig. 6) Re-injection of produced water is the most common disposal method in U.S., and its cost is evaluated to be less than $2/bbl.[12-14] Yet, the cost of produced water treatment varies much depending on regions, as shown in SI-Fig. 6. One of the reasons for such difference in disposal cost is transportation (*i.e.* trucking). For example, Marcellus shale play (in Pennsylvania) has no choice but to truck the waste water to the nearest available injection wells in Ohio, creating conditions for more

5. Designing a multi-stage operation based on the bifurcated ICP desalination

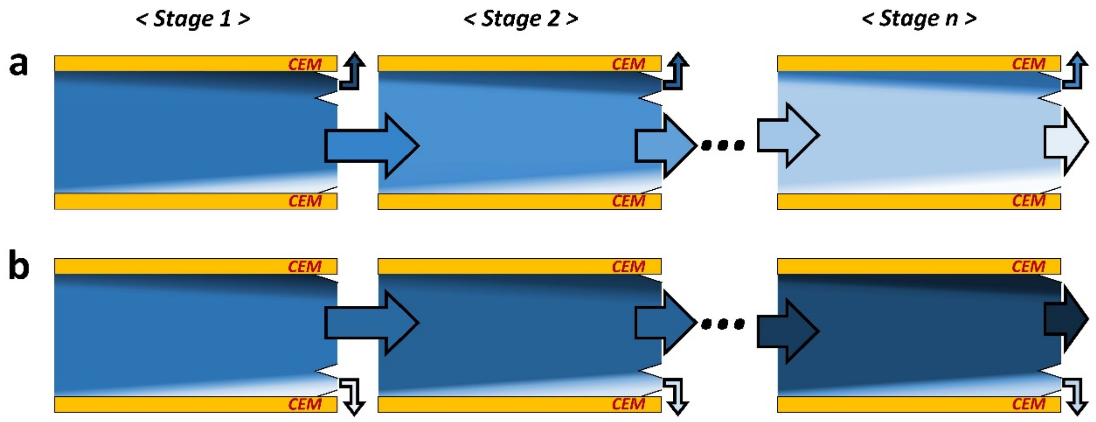

SI-Fig. 7. Schematic illustration of bifurcated ICP strategies for multi-stage desalination. (a) Serial extraction of concentrate stream and (b) dilute stream, respectively. In both cases, one of two streams in each stage is extracted out and another stream is fed into next stage as feed solution.

In addition to trifurcated ICP, bifurcated ICP with two output stream (dilute, concentrate) also could be used for multi-stage desalination. Although basic idea of eliminating incremental amount of salts in each stage remains the same as trifurcated ICP, gradual change of feed salinity would be a prominent concern compared to the trifurcated ICP.

5.1 Serial extraction of concentrate stream

SI-Fig. 7a shows bifurcated ICP for multi-stage operation by the serial extraction of concentrate stream. One can extract small amount of concentrate stream and deliver large amount of dilute stream to the feed flow in the next stage. After processing all stages, dilute stream in the last stage can be obtained as final desalted water, with much more

dilute concentration than feed water. Since the feed concentration gradually increases in each stage, EPIR for each stage would gradually increases, while it is easy to acquire highly pure water finally. In terms of EPIR based on the Fig. 3a in the manuscript, this strategy is appropriate for highly concentrated brine feed water. (> 1M NaCl) Another characteristic is that overall recovery would be decreasing when stage is added and thus more stage bring about higher water purity, but higher average EPIR and lower recovery.

5.2. Serial extraction of dilute stream

SI-Fig. 7b shows bifurcated ICP for multi stage operation by serial extraction of dilute stream. In contrast to the strategy shown in SI-Fig. 7a, one can extract small amount of dilute stream and deliver large amount of concentrate stream to the feed flow in next stage. Dilute streams in every stage are final desalted water that we want to obtain and concentrate stream in the latest stage would be brine waste. Since the feed concentration in each stage gradually increases, EPIR in each stage would gradually decreases while it is difficult to acquire final desalted water with high purity (diluted stream gets desalted only once). In terms of energy efficiency, this strategy is appropriate for brackish water. (< 0.5M NaCl) Overall recovery would be increasing when stage is added and thus more stage bring about lower average EPIR and higher recovery, but low water purity in produced water treatment.[12]

6. Suspended solids deposited on the membrane

Since the surface of CEM and most of suspended solids are negatively charged, there would be less interfacial interactions due to the high electrostatic repulsion when they approach to a close. However, high bulk concentration (which reduces thickness of electric double layer and the electrostatic force) and short distance (which enhances van der Waals force) could facilitate the aggregation of aqueous dispersions according to DLVO theory.[15] In case of ICP desalination, high bulk salinity and the densely populated particles near the CEM can considerably enhance the interfacial interactions between the particles and the CEM surface which potentially brings about severe membrane fouling and degradation. This phenomenon also happens in the electrodialysis when he treats the feedwater containing colloidal particles. In order to clear these aggregated colloidal particles from the membrane surface, reversal of the DC electric field has been adopted (EDR: electrodialysis reversal) since reversed electric driving force removes deposited colloids back into the bulk stream.[16,17] Based on these previous efforts, we have observed massive colloidal particles removed from the CEM surface in the ICP device by applying reversed electric field. (See SI movie 3) As shown in the movie, bunch of particles (crude oil emulsion, used in Figure 7c) come off the CEM surface immediately after reversed electric field is applied. Based on the results, it is reasonable to suggest the ICP desalination operated via periodic reversed electric field (ICP reversal, See SI-Fig. 8) in order to mitigate membrane fouling/degradation when treating large amount of bio-contaminants and colloidal particles.

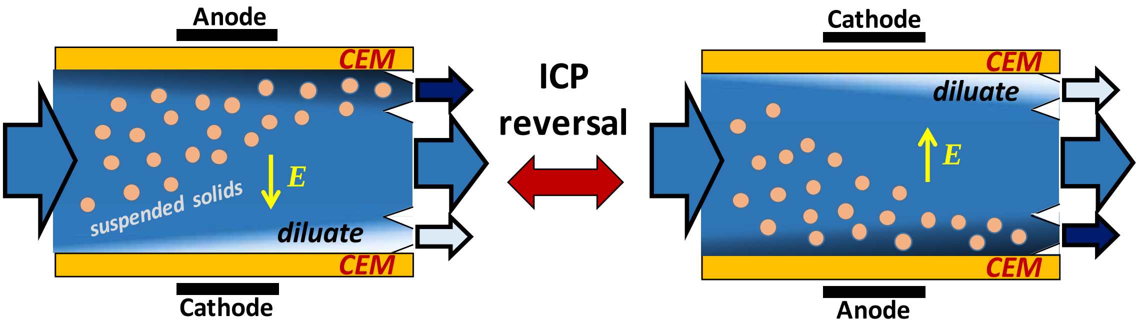

SI-Fig. 8. Proposed strategy to mitigate membrane contamination by suspended solids: ICP reversal is operated via periodic reversal of electric field.

It has to be noted that ICP reversal has two different features compared to the electrodialysis reversal. First, CEM interface where the particles are deposited always becomes dilute stream once the reversed electric field is applied. Lowering bulk concentration accelerate in separation of the particles from the CEM surface because it can reduce interfacial interaction based on the DLVO theory. Secondly, ICP reversal should be more easy to remove the colloidal particles from the membrane surface rather than electrodialysis reversal because the electrodialysis has high electrostatic attraction of colloidal particles to specific ion exchange site in the AEM while ICP does not employ AEM.

7. Studies on pH changes adjacent to the AEM and CEM

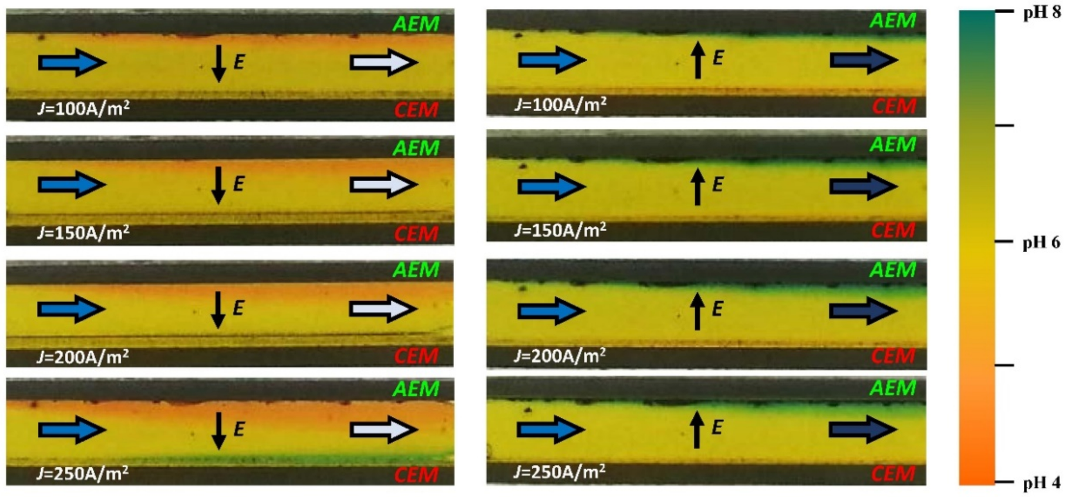

SI-Fig. 9. (a) Observation of pH change near the membrane depending on the applied current density in ED system. Images on left and right side show dilute and concentrate stream, respectively.

We observed real-time pH change near AEM and CEM while increasing the current density (0~250A/m^2) using 0.1M NaCl solution. (SI-Fig. 9) The limiting current density in 0.1M NaCl is ~100A/m^2 as shown in voltage-current response. The pH change near the AEM was observed from the limiting current density. The pH change near CEM was not observed until one reaches the current density of 250A/m^2, which could lead to better resistance to scaling than AEM. Moreover, we found colloidal particles agglomerated and adhered to the AEM surface while CEM surface remained clean for a long-term operation, presumably because of generally negative zeta-potential assumed by colloids. We also observed that, in higher concentrations (> 0.1M), pH change tends to be minimized both near AEM and CEM.

8. Removal of monovalent vs. divalent

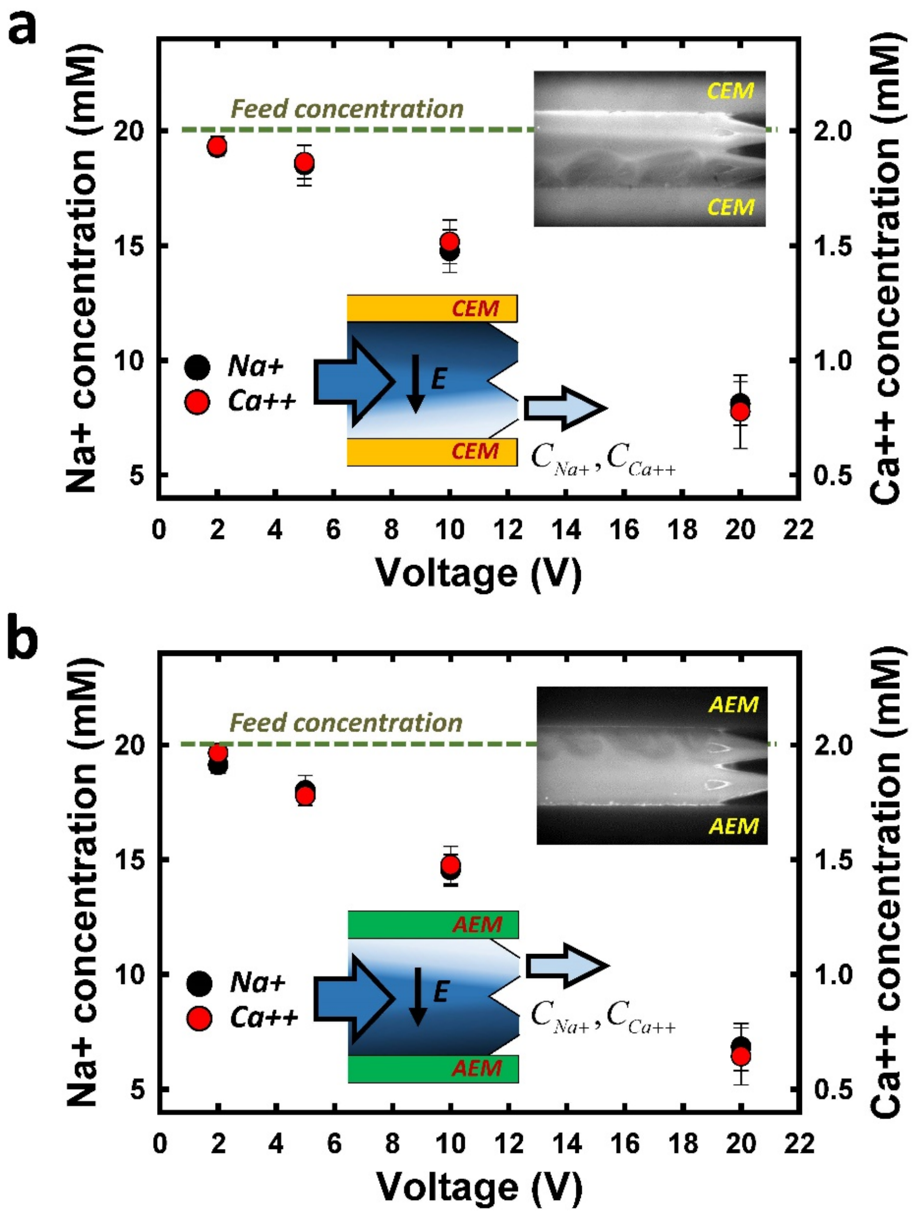

SI-Fig. 10. Concentration change of sodium and calcium ions in (a) ICP-CEMs and (b) ICP-AEMs by analyzing dilute output flow.

We prepared bifurcated ICP-CEMs / ICP-AEMs devices with 1.5mm of inter-membrane distance, 0.6mm of channel depth, and 20mm of channel length. Feed solution contained 20mM NaCl and 2mM $CaCl_2$, simulating typical conditions often found in natural feedwater where NaCl is the majority ion species while Ca^{++} ions are of minority carriers. A certain volume (0.6ml) of dilute output stream after ICP desalination were collected at the outlet each for 20min (under 60 $\mu\ell$/min of dilute flow rate), and dried out to remove the solvent overnight. 2wt. % of HNO_3 solution was added to the solute and the final samples were loaded into the ICP-AES equipment (Activa-S, Horiba Co., Japan) for measuring quantities of sodium and calcium ions post-processing. Final concentration of sodium and calcium in original samples was calculated from the measured values, and the voltage shown in SI-Fig. 10 was measured between anode and cathode.

List of Movies

SI movie 1. Visualization of salt rejection in multi-stacked ICP desalination

SI movie 2. Simultaneous removal of crude oil emulsion as well as salts

SI movie 3. Observation of crude oil emulsions detached from the CEM surface by applying reversed DC electric field

References

1 Nikonenko, V. V. *et al.* Desalination at overlimiting currents: State-of-the-art and perspectives. *Desalination* **342**, 85-106 (2014).

2 Hays, J. Iowa's first electrodialysis reversal water treatment plant. *Desalination* **132**, 161-165 (2000).

3 McGovern, R. K., Weiner, A. M., Sun, L., Chambers, C. G. & Zubair, S. M. On the cost of electrodialysis for the desalination of high salinity feeds. *Applied Energy* **136**, 649-661 (2014).

4 Sajtar, E. T. & Bagley, D. M. Electrodialysis reversal: Process and cost approximations for treating coal-bed methane waters. *Desalination and Water Treatment* **2**, 284-294 (2009).

5 Electric power monthly with data for october 2014. *U.S. Energy Information Administration* (2014).

6 Turek, M. Cost effective electrodialytic seawater desalination. *Desalination* **153**, 371-376 (2003).

7 Gregory, K. B., Vidic, R. D. & Dzombak, D. A. Water management challenges associated with the production of shale gas by hydraulic fracturing. *Elements* **7**, 181-186 (2011).

8 Slutz, J., Anderson, J., Broderick, R. & Horner, P. in *APPEA International Conference on Health, Safety, and Environment in Oil and Gas Exploration and Production, Perth, Australia.* 11-13.

9 Hayes, T., Severin, B. F., Engineer, P. S. P. & Okemos, M. Barnett and Appalachian Shale water management and reuse technologies. *Contract* **8122**, 05 (2012).

10 Horner, P., Halldorson, B. & Slutz, J. A. in *SPE Annual Technical Conference and Exhibition.* (Society of Petroleum Engineers).

11 Boschee, P. Produced and Flowback Water Recycling and Reuse: Economics, Limitations, and Technology. *Oil and Gas Facilities* **3**, 16-21 (2014).

12 Rassenfoss, S. From flowback to fracturing: Water recycling grows in the Marcellus Shale. *Journal of Petroleum Technology* **63**, 48-51 (2011).

13 Drewes, J. E. *et al.* An Integrated Framework for Treatment and Management of Produced Water. *Research Partnership to Secure Energy for America* (2009).

14 Stepan, D. *et al.* (Grand Forks, ND: Energy & Environmental Research Center, 2010).

15 Incorporation, Z. M. Zeta Potential: A Complete Course in 5 Minutes. *Zeta-Meter, Inc* **765** (2005).

16 Valero, F., Barceló, A. & Arbós, R. Electrodialysis technology-theory and applications. *Desalination, trends and technologies* **28**, 3-20 (2011).

17 Allison, R. P. in *American Water Works Association Membrane Technology Conference. Orlando, Florida.*
